# A New Approach for Multimodal Usage of Gene Expression and Its Image Representation for the Detection of Alzheimer’s Disease

**DOI:** 10.3390/biom13111563

**Published:** 2023-10-24

**Authors:** Umit Murat Akkaya, Habil Kalkan

**Affiliations:** Department of Computer Engineering, Gebze Technical University, 41400 Gebze, Turkey; umit.akkaya@gtu.edu.tr

**Keywords:** data fusion, dementia, deep learning, multimodal, Alzheimer’s disease, linear discriminant analysis

## Abstract

Alzheimer’s disease (AD) is a complex neurodegenerative disorder and the multifaceted nature of it requires innovative approaches that integrate various data modalities to enhance its detection. However, due to the cost of collecting multimodal data, multimodal datasets suffer from an insufficient number of samples. To mitigate the impact of a limited sample size on classification, we introduce a novel deep learning method (*One2MFusion*) which combines gene expression data with their corresponding 2D representation as a new modality. The gene vectors were first mapped to a discriminative 2D image for training a convolutional neural network (CNN). In parallel, the gene sequences were used to train a feed forward neural network (FNN) and the outputs of the FNN and CNN were merged, and a joint deep network was trained for the binary classification of AD, normal control (NC), and mild cognitive impairment (MCI) samples. The fusion of the gene expression data and gene-originated 2D image increased the accuracy (area under the curve) from 0.86 (obtained using a 2D image) to 0.91 for AD vs. NC and from 0.76 (obtained using a 2D image) to 0.88 for MCI vs. NC. The results show that representing gene expression data in another discriminative form increases the classification accuracy when fused with base data.

## 1. Introduction

Alzheimer’s disease (AD), which is a class of dementia, represents a significant and escalating global health problem that is characterized by its profound impact on cognitive function and quality of life [1]. Alzheimer’s disease (AD), which leads to cognitive impairment, is among the most common neurodegenerative conditions affecting elderly individuals. It is an irreversible and progressive disorder marked by symptoms such as memory loss, cognitive decline, and emotional disturbances [2]. According to the Office for National Statistics (ONS), ‘dementia and Alzheimer’s disease’ emerged as the primary cause of death in 2022 [3].

AD starts with a cognitive decline stage, which is called a mild cognitive stage (MCI), that may lead to AD within years. Traditionally, AD diagnosis has largely relied on clinical evaluation, cognitive testing, and neuroimaging, often detecting the disease at advanced stages when irreversible neuronal damage has already occurred. The multifaceted nature of AD, which involves genetic, environmental, and molecular factors, necessitates a more comprehensive and precise diagnostic strategy. Gene expression analysis, which provides insights into the activity of thousands of genes within cells and tissues, holds the potential to illuminate the subtle molecular changes occurring during the prodromal and early stages of AD.

Machine learning (ML) algorithms have frequently been used to estimate AD or MCI by using the data obtained from samples. However, ML algorithms, especially deep learning (DL) models, suffer from the problem of high dimension, low sample size (HDLSS), which is also valid for gene expression datasets where thousands of genes are usually obtained for tens/hundreds of subjects [4,5]. To eliminate the HDLSS problem, researchers represent the gene expression data of subjects in a low dimension by either extracting new features or by selecting the differentially expressed genes (DEGs) using ML algorithms [6].

DL, which is a subbranch of ML, allows for the elimination of the feature extraction steps and extracts discriminative features in the hidden layers of the network. DL is an effective tool for creating estimation or classification models for AD by using data from different modalities (MRI, PET, fMRI, EEG etc.). For example, Tian et al. [7] used retinal images measured using optical coherence tomography (OCT) and proposed a deep learning-based image quality detector and feature extractor for AD classification. Similarly, AlSaaed et al. [8] and Saratxaga et al. [9] used DL on a brain MRI dataset including 741 subjects obtained from the OASIS-1 [10] and OASIS-2 [11] datasets. The recent progress on in DL leads has led to efficient model generation which combines multimodal data for AD classification. Mirabnahrazam et al. [12] used MRI data and genetic information from 521 subjects for the estimation of AD at four different levels. They processed the MRI data and obtained volumetric features and 521,014 single nucleotide polymorphisms (SNP) and selected the most discriminative 17 features for classification. Chen et al. [13] used MRI, cerebrospinal fluid (CSF), and fluorodeoxyglucose-positron emission tomography (FDG-PET) datasets from the Alzheimer’s Disease Neuroimaging Initiative (ADNI) for AD detection. They processed 34 AD, 87 MCI, and 63 CN (cognitively normal) samples. Chen et al. [13] represented each dataset in vectoral form using a feature extraction step in which they evaluated voxel and cortical thickness from the MRI and FDG-PET images and fused the feature vectors pairwise before classification. In another study, Chen et al. [14] used PET, MRI, clinical, and demographic data from 227 subjects from the ADNI dataset including 45 AD, 92 stable MCI (sMCI), 33 progressive MCI (pMCI), and 57 NC. They represented the MRI and PET images using a convolutional block and fused the features obtained from the PET and MRI images with the features from medical (MED) and clinical data using a channel attention (CA) and spatial attention (SA) mechanism.

Although there are plenty of datasets for AD, many of them include single-modal data [8,15,16]. In general, a dataset containing multimodal data [12,13,14] has a limited number of subjects compared to a dataset containing single-modal data [8,15,16]. Due to the low number of subjects in multimodal datasets, some researchers transform the acquired data into a different form for better representation of the samples. In that scope, Sharma et al. [17] proposed a method for converting mRNA data into a 2D image using t-SNE [18] and kernel principal component analysis (kPCA) and then feeding the obtained image into a CNN for classification. However, t-SNE and kPCA are unsupervised techniques for dimension reduction and they do not consider discriminative genes while mapping. However, Kalkan et al. [19], used linear discriminant analysis (LDA) to transform the gene expression data into a 2D image to be able to use them in CNN algorithms. The supervised properties of LDA led discriminative genes to be within immediate vicinities on 2D images and this was reported to increase the accuracy of the CNN classifier. The studies indicate that representing gene expression in other forms (e.g., a 2D image) can lead to higher classification accuracies.

In this study, we hypothesized that representing gene expression data as 2D images with high discrimination content and fusing these images with gene expression data after feeding them into DL models would improve classification accuracy. Within the scope of this hypothesis, a novel data fusion approach was proposed to fuse the one-dimensional gene expression data and the 2D image that is obtained from the same gene expression dataset. The proposed method first selects the DEGs and represents the DEGs vector as a 2D image which maximizes the distance between classes and minimizes the variance within classes. Then, an FNN and CNN network are trained using the DEGs vectors and the 2D image representation of the DEGs vectors, respectively. The outputs of the models are then fused before being fed into a joint deep model which performs the binary classification.

## 2. Materials and Method

### 2.1. Dataset

Three sets of Alzheimer’s disease study datasets were obtained (GSE63060 [20], GSE63061 [20], GSE140829 [21]) from the National Library of Medicine National Center of Biotechnology Information (NCBI) database (Table 1). Normalized gene expression datasets were combined to create a total of 1262 samples. These samples encompass individuals from Alzheimer’s disease (AD), mild cognitive impairment (MCI), and normal control (NC) categories. Supplementary demographic information is provided in Table S2.

Three datasets were first standardized individually and then combined according to their respective groups (AD, MCI, and NC) using the min–max normalization technique. This reduced gene expression values to a range between 0 and 1. The GSE63060, GSE63061, and GSE140829 normalized datasets have 29,958, 24,900, and 15,987 probes, respectively. Notably, 11,618 shared probes were found in all three datasets.

### 2.2. FNN-Based Classification of Gene Expression

An FNN, also known as a multi-layer perceptron (MLP), is one of the simplest and most common types of artificial neural networks [22]. It consists of an input layer, one or more hidden layers, and an output layer. Information flows in one direction, from the input layer to the output layer, with no loops or cycles. The layers may include multiple neurons and each neuron is represented by a function which collects information from the connected neurons in the previous layer. The FNN updates the weights between neurons to decrease the estimation/classification error at the output neuron(s).

**Figure 1:**
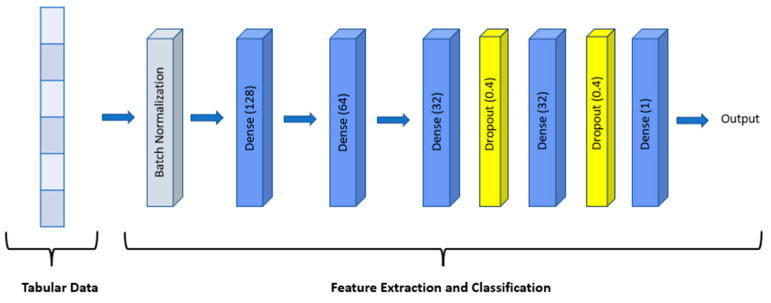
Tabular data trained with a sequential FNN model which contains dense layers of 128 and 64 and two dense layers of 32 that are followed by two dropout layers.

In this study, an FNN model with 1,545,129 parameters was used for classification (Figure 1). All gene expression data were fed into the model for feature extraction and classification. The model consists of dense layers which have 128, 64, 32, and 32 units, respectively. Dropout layers and L1 and L2 regularizers were added after the last two dense layers for regularization and to prevent overfitting of the model.

### 2.3. Image-Based Representation of Gene Expression and CNN-Based Classification

A considerable number of genes are expected to have minimal significance, and their incorporation could introduce a substantial number of irrelevant features or noise into the architecture of the CNN. To mitigate this issue, a preemptive dimension reduction step was taken before initiating the image-based transformation. The irrelevant genes were removed prior to completing the image-based modification using the LASSO (least absolute shrinkage and selection operator) regression approach [23], which performs feature selection and regularization to enhance classification accuracy.

**Figure 2:**
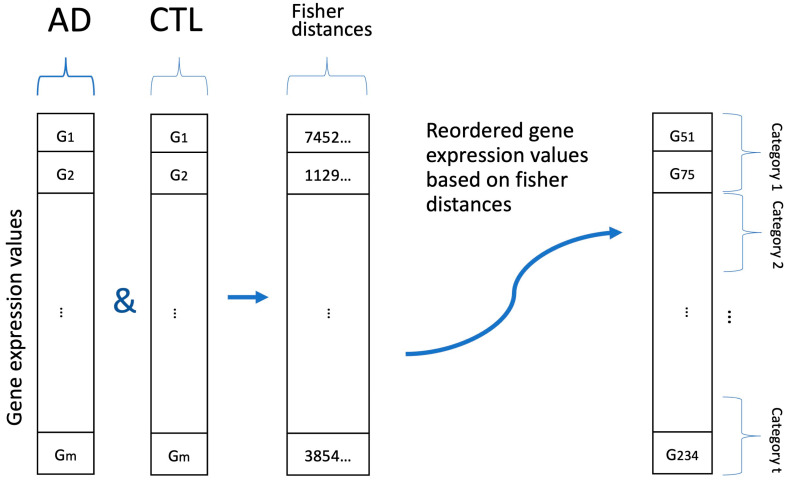
This graphic comprises two sections, one depicting a gene expression array for AD and the other for CTL (control) classes. The third section displays the Fisher distances calculated for each gene and the final section illustrates how genes are categorized based on these Fisher distances [19].

The image-based representation method was executed in two stages. Initially, genes were categorized based on their ability to distinguish between disease and control conditions. Subsequently, their discriminatory capabilities were employed to visualize them in 2D images. In the initial phase, the discriminative potential of the genes was assessed using the Fisher distance [24]. The Fisher distance metric was chosen because it optimizes the separation between classes while minimizing variance within each class. Subsequently, the genes were grouped and labelled based on their Fisher distances. Each category was assigned an equal number of samples (Figure 2).

**Figure 3:**
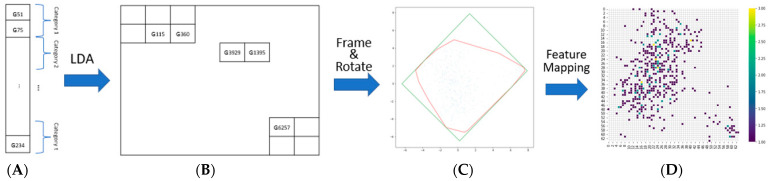
LDA is used to locate the genes in the 2D picture. (**A**) depicts the gene classification. The position of the genes in a 2D picture produced by LDA is shown in (**B**). The minimal rectangle produced from (**A**) is (**C**). (**D**) represents the gene expression at the relevant site [19].

Linear discriminant analysis (LDA) is utilized to map every gene into a 2D space in this study. LDA, a supervised machine learning technique, is employed to enhance the distinction between various gene categories and maximize classification accuracy. Its purpose here is to position genes from the same categories close to each other in the 2D space. However, there might be empty areas on the 2D map where no genes are assigned. To minimize these gaps and create a more compact image, convex hull algorithms [17] were applied to determine the smallest rectangle which is then rotated to align with the 2D coordinate system. Each non-zero pixel in the final 2D image represents a gene’s location in the sequence. Using this location data, we position each gene in its corresponding spot on the 2D image (Figure 3). Adjusting the image’s resolution can either bring gene expression values closer together or spread them apart. Opting for lower resolution may lead to multiple genes mapping to the same location in the 2D image. Consequently, gene expression values that project to the same location are averaged and this average value is placed in the corresponding spot.

**Figure 4:**
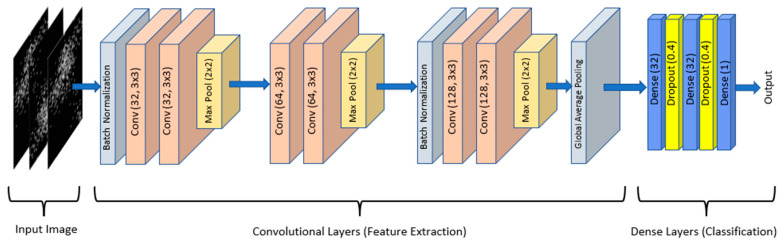
The first two convolutional layers contain 32 filters, the third and fourth layers have 64 filters, and the last two layers have 128 filters. Convolutional layers are followed by dense and dropout layers for classifying the features which are learned and generated at convolutional layers [19].

A CNN is a deep learning architecture designed primarily for tasks involving grid-like data such as images and, more recently, sequential data like text and speech [25]. CNNs have been instrumental in revolutionizing computer vision tasks like image classification, object detection, and image segmentation. In contrast to an FNN which works on features extracted from the data, a CNN model includes convolutional layers whose purpose is to extract discriminative features for the learning operation. So, a CNN eliminates the necessity of a feature extraction step in machine learning algorithms which is a tedious process, especially in image classification problems. In this study, a CNN model (Figure 4) was used for the classification of 2D gene images.

This CNN model with 292,493 parameters, as depicted in Figure 4, was employed as the architecture for the analysis. This CNN consists of six convolutional layers, organized in pairs followed by a pooling layer. The convolutional layers are then followed by two dense layers with dropout and classification layers with a sigmoid function.

### 2.4. One2MFusion

Multimodal machine learning is an advanced method that combines data from several sources or modalities (text, pictures, audio, sensor data, etc.) to improve the prediction power and robustness of machine learning models [26,27,28,29]. This paradigm has received a lot of interest in a variety of fields ranging from computer vision and natural language processing to healthcare and finance [30,31]. In recent years, DL contributed to multimodal machine learning data at different levels. The fusion is usually performed at the data level (before the deep network), at the output level (before the decision layer), or at the model level (within trained layers).

**Figure 5:**
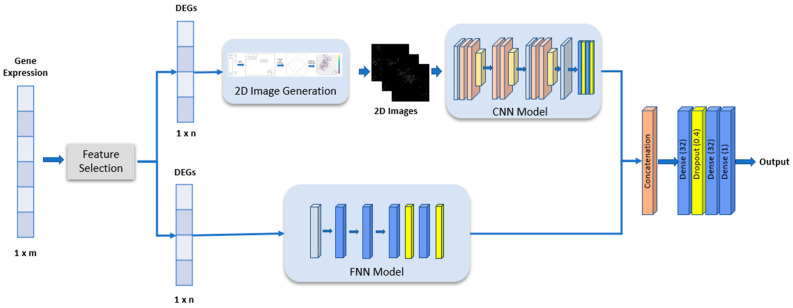
Overall architecture of the proposed *One2MFusion* model.

In this study, we proposed a multimodal deep learning model, *One2MFusion*, which fuses the gene expression data in tabular form and the 2D representation of the tabular gene expression data for classification (Figure 5).

The proposed approach initially reduces the dimensionality of gene expression data (*∈R^m^*) to a lower dimension (*∈R^n^*) by selecting a subset of DEGs using LASSO regression. The gene expression array containing the chosen genes follows two distinct paths for feature extraction: FNN and CNN.

In the lower layer, the FNN model (depicted in Figure 1) is employed to process the tabular data represented by the DEGs array, resulting in a feature vector of size t (<n). In the parallel path (top layer), the DEGs array undergoes a transformation into 2D images, as explained in Section 2.3. These generated images are then utilized to train the proposed CNN model, as illustrated in Figure 4. Notably, the dimension of the fully connected layer in the CNN model is restricted to t, aligning it with the size of the output layer from the FNN model.

The feature vectors (*∈R^t^*) generated by the CNN and FNN models are subsequently merged at a concatenation layer. The merging is basically performed by placing the vectors of the output layers of the CNN and FNN into a single vector (*∈R^2t^*). This fused vector is then passed into two dense layers, each comprising 32 units, with a dropout layer (dropout rate of 0.4) in between. Finally, the binary classification layer is applied to make predictions based on the processed features.

During the training process, a callback object was implemented for all models. This callback monitored the loss after the initial 250 epochs, halted training, and saved the model if the loss did not decrease for the subsequent 10 epochs. To mitigate overfitting, the last two dense layers of the FNN and CNN models are accompanied by L1 and L2 regularization (L1 = 1 × 10^−5^, L2 = 1 × 10^−4^), followed by dropout layers (0.4). The choice of activation functions was *Relu* for the dense layers and Sigmoid for the output layers for all models. For the models, binary cross entropy was used as the loss function and an Adam optimizer was employed with a learning rate of 1 × 10^−4^. All models were implemented using Python (Version 3.7.13, Python Software Foundation, Wilmington, DE, USA) with the Keras library (Version 2.8.0, init. Author François Chollet), utilizing TensorFlow as the backend (Version 2.8.2, Google Brain Team, Mountain View, CA, USA). All computations and evaluations were conducted on Google Colab (Google Brain Team, Mountain View, CA, USA). Experiments were conducted on a Colab Tesla T4 (equipped in Amazon EC2 G4 instance) GPU. Performance metrics were collected using Keras metrics except area under curve (AUC) and receiver operating characteristics (ROC) which were collected using Scikit-learn (Version 1.3.0) metrics [32].

## 3. Results

Although it is possible to use the 11,168 common genes for classification, selecting the most discriminative genes allowed us to represent the subjects with fewer but more representative genes. The discriminative genes were selected using the LASSO regression method. In the LASSO method, the number of selected genes is related to the *λ* parameter. Experimentally, various values between 1 × 10^−1^ and 1 × 10^−4^ were assigned to the *λ* parameter and the classification tasks were performed. However, the best classification outputs were obtained with the selected genes when *λ* was selected as 1 × 10^−6^ Setting *λ* as 1 × 10^−6^ resulted in 488, 492, 420, 492, and 401 gene expressions among 11,168 gene expression for AD vs. NC, AD vs. MCI, AD vs. (MCI + NC), and (AD + MCI) vs. NC, respectively. The dataset was divided into five subgroups for five-fold cross validation and the output of the folds were averaged and presented. The five-fold cross validation divides the dataset into five disjoint subsets and repeats the training five times by leaving out one subset which is reserved for testing. The trained models are evaluated for accuracy, sensitivity, recall, F1-score, ROC, and AUC. For fair comparison, the state-of-the-art methods were also trained and tested within the same cross validation.

### 3.1. D Representation of Genes

**Figure 6:**
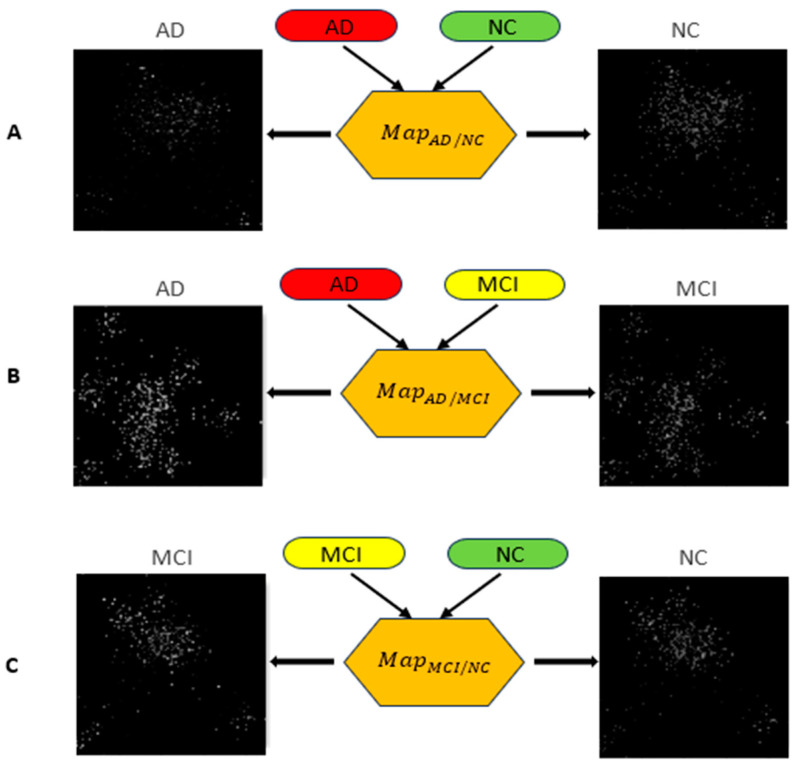
2D image representation of randomly selected AD, MCI and NC samples’ gene representation using three pairwise mappings (*Map_AD/NC_*, *Map_AD/MCL_*, and *Map_MCI/NC_*). Row (**A**) shows the generated AD and NC images of an AD and NC gene expression; row (**B**) shows the generated AD and MCI images of an AD and MCI gene expression, and row (**C**) shows the generated MCI and NC images of an MCI and NC gene expression.

The proposed LDA-based image generation involves dividing the subjects into categories using the Fisher distance. In this study, the genes were divided into 7, 9, 11, 13, 15, and 17 categories, and the image-based classification was performed. Based on Kalkan et al. [19], 15 was selected as the number of categories for LDA-based mapping. For AD vs. NC, AD vs. MCI, AD vs. MCI + NC, and AD + MCI vs. NC pairwise classification, a different image mapping (*Map_AD/NC_*, *Map_AD/MCL_*, and *Map_MCI/NC_*) is generated. Figure 6 shows the generated 2D images for three randomly selected samples (AD, MCI, and NC) using the LDA-based mapping.

### 3.2. Classification

In the proposed *One2MFusion* model, the deep neural networks which were used for learning the gene expression, 2D image representation, and fusion were trained with a batch size of 30, and the models were trained until an early stopping criterion which stops learning when ten successive non-improvements in training loss occur. The *One2MFusion* method was used for pairwise classification and the highest AUC value of 0.91 was obtained for AD vs. NC classification (Table 2). The proposed method gave the second highest AUC of 0.88 for AD vs. MCI classification. For the MCI (which is regarded as an intermediate state for AD) samples, it was questioned from the perspective of classification performance whether they should be added to the AD or NC classes for binary classification. It should be stated that the MCI is regarded as an intermediate state for AD [33]. This statement was also observed in the classification results in Table 2 where joining the MCI samples with NC (third row) leads to the same classification accuracy compared to the case where MCI samples are joined with the AD group (fourth row). In addition, the proposed model gave an AUC value of 0.85 for MCI vs. NC which indicates the high discrimination between the NC and MCI samples.

The *One2MFusion* method combines two deep models, namely an FNN and CNN, which were trained separately using either gene expression data or 2D image representations derived from the same gene expressions. To assess the impact of *One2MFusion* on classification outcomes, the deep models trained on either gene expression data (FNN model) or 2D image representations (CNN model) were compared to the *One2MFusion* method in terms of AUC values. The results presented in Table 3 reveal that the CNN model trained on 2D image representations consistently yielded higher AUC values compared to the FNN model trained solely on gene expression data across all pairwise classifications. However, the fusion of these deep models, as proposed by the *One2MFusion*, leads to a significant enhancement in classification performance measured by AUC. Remarkably, in the context of the AD vs. NC classification task, the *One2MFusion* method achieved an AUC value of 0.91, surpassing the AUC values of 0.52 and 0.86 obtained when utilizing gene expression data or 2D image representations alone, respectively.

Using the same processing sources on Google Colab, the proposed fusion model exhibited notable processing efficiency and required less time for training than the FNN and CNN models while achieving improved classification results in that the FNN model completed training in 7 min and 46 s, the CNN model required 16 min and 27 s for training, and the *One2MFusion* model completed training in 14 min and 38 s. 

**Figure 7:**
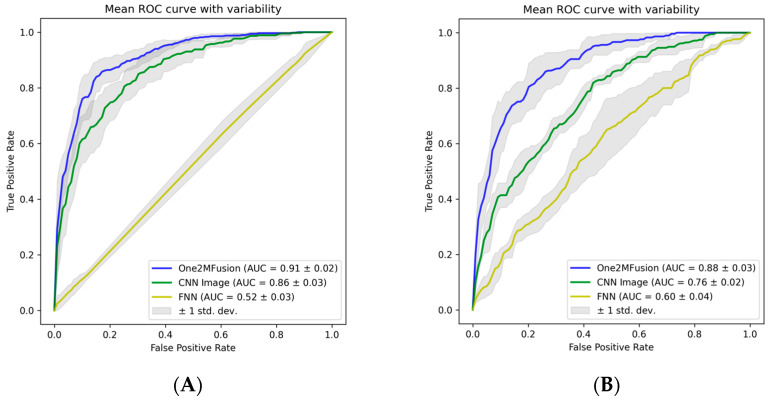
ROC of the model obtained using only gene expression data or 2D images generated from gene expression data or using them both in the *One2MFusion* method for (**A**) AD vs. NC and (**B**) AD vs. MCI classification.

The effect of fusion on AD vs. NC and AD vs. MCI is also presented on the mean ROC curve (Figure 7). It is observed in Figure 7 that the *One2MFusion* method results in a better ROC curve when gene expression data or 2D image representations are used individually.

Using machine learning techniques, several studies have been performed on gene expression classification. Pairwise classification was compared with alternative studies in the literature. To make a fair comparison, the combined dataset from GSE63060, GSE63061, and GSE140829 was used with publicly available codes [17,19,34,35] using the same five-fold cross validation.

In their studies, El-Gawady et al. [34] and Guckiran et al. [35] process gene expression data by first selecting the most discriminative ones and then performing classification using SVM. However, Kalkan et al. [19] and Sharma et al. [17] convert gene expression data into a 2D image and perform classification using a CNN. Extensively, the developed *One2MFusion* method fuses the gene expression array and the 2D images generated from the gene expressions. It is observed in Table 4 that the method by Sharma et al. [17] outperforms the array-based [34,35] methods, which reveals the effect of using a CNN on 2D representations of genes. In addition, Kalkan et al. [19] generated 2D images using a supervised approach and they outperformed Sharma et al. [17] who generated the 2D images in an unsupervised manner.

However, the current method, *One2MFusion*, led to the highest accuracy (0.83) and AUC (0.91) among the other methods for AD vs. NC classification.

Pairwise classifications were also conducted for AD vs. MCI (Table 5) and MCI vs. NC (Table 6) to compare the performance of the same set of studies. The results indicated more significant improvements than those observed for AD vs. NC classification. Once again, the One2MFusion method outperformed the other techniques, achieving the highest accuracy (0.79) and AUC (0.88) for AD vs. MCI as well as an accuracy of 0.77 and an AUC of 0.85 for MCI vs. NC.

## 4. Discussion

Although multimodal data hold promise for early disease diagnosis, they present significant challenges in terms of labor and cost of data collection [36]. As a result, research in the context of multimodal data for Alzheimer’s disease (AD) diagnosis often relies on a limited number of samples [7,8,13,14].

Gene expression data obtained from blood samples are considered a valuable resource for AD diagnosis. However, gene expression datasets frequently suffer from the high dimension, low sample size problem, typically featuring thousands of genes for only tens or hundreds of samples, which is suboptimal for deep learning studies.

In this study, we have introduced an innovative approach (called *One2MFusion*) for fusing multimodal data, specifically gene expression and 2D image representations of these genes, utilizing deep learning techniques. Our study stands out by transforming gene expression data into another modality and fusing them to reveal more discriminative features for classification. Essentially our approach treats 2D image representations as a new set of samples for classification improvement. Rather than introducing a different modality from an external data source, we leverage the same dataset and transform it into an additional modality. To construct a gene expression dataset, we merged three different datasets (GSE63060, GSE63061, and GSE140829), considering the common genes, and generated 2D image representations of these samples using an LDA-based dissimilarity approach. We conducted five-fold cross validation for five distinct pairwise classifications: AD vs. NC, AD vs. MCI, AD vs. (MCI + NC), and (AD + MCI) vs. NC. The *One2MFusion* method, which integrates gene expression data and 2D image representations of these genes, was compared to deep learning methods that employed either gene expression data or 2D image representations.

Our experimental results reveal that 2D image representations of genes, as previously reported in Kalkan et al. [19], consistently yield higher classification accuracies across all pairwise classifications compared to using only gene expression data. However, the fusion of data from these two different modalities significantly enhances classification accuracies. For instance, the AUC for AD vs. MCI classification increased from 0.76 to 0.88, AUC for MCI vs. NC classification improved from 0.77 to 0.85, and AUC for AD vs. NC classification improved from 0.86 to 0.91.

We also conducted a comparison between the proposed *One2MFusion* method and state-of-the-art techniques [17,19,34,35], all of which provide publicly available code. This comparison involved using the same combined dataset and cross validation methodology, specifically focusing on the AD vs. NC classification task. Notably, the *One2MFusion* method outperformed the referenced studies, achieving an AUC of 0.91, in contrast to their respective AUC scores of 0.77 [34], 0.73 [35], 0.70 [17], and 0.85 [19].

This study aimed to represent gene expression data as 2D images with high discrimination content and to fuse these images with gene expression data to obtain higher classification accuracies. The experiments revealed the contribution of generated 2D images to AD classification either used individually or fused with source data. It is important to highlight that the developed method, accompanied by publicly accessible source code, can be readily applied to gene expression datasets for various diseases, which will be our focus in future studies. The developed method can also be applied to other types of tabular data. However, it should be noted that tabular data with a relatively low dimension compared to gene expression datasets may limit the generation of descriptive 2D images for CNNs.

## Figures and Tables

**Table 1:** The content of the studied datasets. The last column is the combination of all datasets.

Groups	GSE63060	GSE63061	GSE140829	Combined Dataset
AD	145	139	198	482
MCI	80	109	124	313
NC	104	134	229	467
Total	329	382	551	1262

**Table 2:** Pairwise classification results of the developed *One2MFusion* method.

Classes	Accuracy	AUC	Precision	Recall	F1-Score
AD vs. MCI	0.79	0.88	0.73	0.75	0.74
MCI vs. NC	0.77	0.85	0.73	0.69	0.71
AD vs. (MCI + NC)	0.76	0.83	0.79	0.83	0.81
(AD + MCI) vs. NC	0.76	0.83	0.69	0.64	0.66
AD vs. NC	0.83	0.91	0.83	0.84	0.83

**Table 3:** Pairwise classification results obtained using deep neural network models which were trained using only gene expression data, only 2D image representations of genes, or both (*One2MFusion*).

Classes	FNN Model (Using Only Gene Expression)	CNN Model (Using Only 2D Image of Gene Expression)	Proposed *One2MFusion*
AD vs. MCI	0.60	0.76	0.88
MCI vs. NC	0.51	0.77	0.85
AD vs. (MCI + NC)	0.50	0.80	0.83
(AD + MCI) vs. NC	0.51	0.80	0.83
AD vs. NC	0.52	0.86	0.91

**Table 4:** AD vs. NC classification results obtained with publicly available studies and our proposed method.

Study	Method	Accuracy	AUC	Precision	Recall	F1-Score
El-Gawady et al. [34]	Multiple Feature Selection + SVM	0.67	0.67	0.67	0.65	0.66
Guckıran et al. [35]	Lasso + SVM	0.73	0.73	0.74	0.71	0.72
Sharma et al. [17]	DeepInsight (tSNE + CNN)	0.68	0.70	0.65	0.75	0.70
Kalkan et al. [19]	LDA-based imaging + CNN	0.81	0.85	0.82	0.79	0.80
Our study	*One2MFusion*	0.83	0.91	0.83	0.84	0.83

**Table 5:** AD vs. MCI classification results obtained with publicly available studies and our proposed method.

Study	Method	Accuracy	AUC	Precision	Recall	F1-Score
El-Gawady et al. [34]	Multiple Feature Selection + SVM	0.61	0.58	0.50	0.46	0.48
Guckıran et al. [35]	LASSO + SVM	0.64	0.63	0.66	0.44	0.53
Sharma et al. [17]	DeepInsight (tSNE + CNN)	0.58	0.60	0.47	0.54	0.50
Kalkan et al. [19]	LDA-based imaging + CNN	0.65	0.62	0.56	0.48	0.52
Our study	*One2MFusion*	0.79	0.88	0.73	0.75	0.74

**Table 6:** MCI vs. NC classification results obtained with publicly available studies and our proposed method.

Study	Method	Accuracy	AUC	Precision	Recall	F1-Score
El-Gawady et al. [34]	Multiple Feature Selection + SVM	0.62	0.60	0.53	0.50	0.51
Guckıran et al. [35]	LASSO + SVM	0.62	0.57	0.43	0.44	0.43
Sharma et al. [17]	DeepInsight (tSNE + CNN)	0.62	0.63	0.54	0.46	0.50
Kalkan et al. [19]	LDA-based imaging + CNN	0.62	0.63	0.53	0.57	0.55
Our study	*One2MFusion*	0.77	0.85	0.73	0.69	0.71

## Data Availability

Publicly available datasets were analyzed in this study. These data can be found here: [https://www.ncbi.nlm.nih.gov/]. The combined data can be found here: [https://www.dropbox.com/scl/fi/84mxumsqhxq2ivb78izp8/GSE_63060_63061_140829.zip?rlkey=wvzspz3cowucyre5vx0np4jeh&dl=0].

## Supplementary Materials

The following supporting information can be downloaded at: https://www.mdpi.com/article/10.3390/biom13111563/s1, Table S1: Alzheimer’s disease detection studies based on gene expression data [19,37,38,39,40,41]; Table S2: Demographic overview of the datasets. The code can be found here: [https://github.com/akkayaumit/One2MFusion-MultiModel-AD-Detection.git].
