# Supplementary material for: A New Approach for Multimodal Usage of Gene Expression and Its Image Representation for the Detection of Alzheimer’s Disease
Source: Biomolecules. 2023 Oct 24;13(11):1563. doi: 10.3390/biom13111563 (PMC10669658; doi:10.3390/biom13111563)
Supplement: Supplementary file 1 [file biomolecules-13-01563-s001.zip › biomolecules-2629839-supplementary.pdf]

Table S1. Alzheimer's disease detection studies based on gene expression data. AUC (Area under the curve); Acc (Accuracy); DNN (Deep Neural Network); SVM (Support Vector Machine; RF (Random Forest); CFG (Convergent Functional Genomics); LASSO (Least Absolute Shrinkage and Selection Operator); PCA (Principal Component Analysis); t-SNE (t-distributed Stochastic Neighbor Embedding); RFE (Recursive Feature Elimination);

Study	Dataset	Number of Samples	Number of Genes	GS Method	Number of Selected Genes	ML Model	Performance
Lee et al. [40]	GSE63060<br>GSE63061<br>ADNI	AD:145, CTL:104 AD:139, CTL:134 AD:63, CTL:136	7584<br>6154<br>3897	CFG	353<br>188<br>922	DNN<br>SVM<br>DNN	AUC: 0.874<br>AUC: 0.804<br>AUC: 0.657
Li et al. [37]	GSE63060 + GSE63061	AD:245, CTL:182	16,928	LASSO	3601	SVM	AUC: 0.859<br>Acc: 0.781
Wang et al. [38]	GSE5281	AD:87, CTL:74	23,643	t-test	1001	SVM	AUC: 0.894
Park et al. [39]	GSE33000 + GSE44770	AD:439, CTL:257	19,488	PCA<br>t-SNE	35<br>35	RF<br>SVM	AUC: 0.531, Acc: 0.624<br>AUC: 0.511, Acc: 0.632
Voyle et al. [41]	GSE63061 + DCR	AD:118, N:118	261	RFE	12	RF	AUC: 0.724, Acc: 0.657
Kalkan et al. [19]	GSE63060 +<br>GSE63061+<br>GSE140829	AD:145, MCI:80, CTL:104<br>AD:139, MCI:109, CTL:134<br>AD:198, MCI:124, CTL:229	11,618	LASSO	488	LDA-based imaging + CNN	AUC: 0.842, Acc: 0.875

Table S2. Demographic overview of the datasets.

	GSE_63060		GSE_63061		GSE_140829	
**Classes**	**N (M/F)**	**Age years+- STDEV**	**N (M/F)**	**Age+- STDEV**	**N (M/F)**	**Age years+- STDEV**
**AD**	145 (46/99)	75.4+-6.58	139 (54/85)	77.89+-6.67	204 (100/104)	73.0+-7.09
**MCI**	80 (41/39)	74.45+- 6.00	114 (46/68)	78.39+-7.38	134 (72/62)	73.2+-6.99
**CTL**	104 (42/62)	72.375+- 6.34	135 (54/ 1)	75.41+-6.17	249 (110/139)	73.6+-6.25
